# Resolution of a T1-Like Bacteriophage Outbreak by Receptor Engineering

**DOI:** 10.1007/s12033-025-01453-1

**Published:** 2025-06-03

**Authors:** Katrina A. Black, Julie V. Nguyen, Jolene R. Ramsey, Jack C. Tovey, Daniel L. Cameron, Jack Alexandrovics, Alisa Glukhova, Anthony T. Papenfuss, Melissa J. Call, Ryland Young, Matthew E. Call

**Affiliations:** 1 The Walter and Eliza Hall Institute of Medical Research, Parkville, VIC Australia; 2 Department of Medical Biology, University of Melbourne, Parkville, VIC Australia; 3 Department of Cellular and Molecular Pharmacology, University of California, San Francisco, San Francisco, CA USA; 4 Department of Biology, Center for Phage Technology, Texas A&M University, College Station, TX USA; 5 Drug Discovery Biology Theme, Monash Institute of Pharmaceutical Sciences, Monash University, Parkville, VIC Australia; 6 ARC Centre for Cryo-Electron Microscopy of Membrane Proteins, Monash Institute of Pharmaceutical Sciences, Monash University, Parkville, VIC Australia; 7 Department of Biochemistry and Pharmacology, The University of Melbourne, Melbourne, VIC Australia; 8 Department of Biochemistry and Biophysics, Center for Phage Technology, Texas A&M University, College Station, TX USA

**Keywords:** Bacteriophage receptor, Bacteriophage resistance, Siphophage, LptD, CRISPR/Cas9, Receptor engineering

## Abstract

Bacteriophage contaminations pose substantial risks to biomolecular production pipelines, and their resolution is especially difficult when the identity of the offending agent is unknown. We recently experienced an outbreak of *Escherichia coli* culture lysis in our Melbourne-based structural biology labs that halted protein production despite our use of T1-resistant (*TonA*/*FhuA*-disrupted) strains. Genetic analysis of the isolated phage yielded a 45,053 bp genome showing 80–90% identity with multiple Rtp-like siphophages, and transmission electron microscopy images were consistent with this classification. Further analysis revealed that our isolate was nearly identical to a highly virulent lytic coliphage MSK, recently isolated in Hangzhou, China, whose host receptor has not been determined. Sequence and structural modelling analysis of its putative receptor-binding protein suggested that its terminal receptor was likely to be LptD, an essential outer membrane protein involved in lipopolysaccharide transport. Based on a recent report of spontaneously arising mutations that blocked infection by other LptD-dependent bacteriophages, we designed a targeted genomic LptD loop deletion that successfully generated resistance to vB_EcoS_OzMSK in *E. coli* BL21(DE3) without apparent detriment to fitness. Here, we report a CRISPR-based, single-plasmid solution that will benefit other labs or facilities experiencing challenges due to LptD-dependent lytic phage outbreaks.

**Supplementary Information:**

The online version contains supplementary material available at 10.1007/s12033-025-01453-1.

## Introduction

Bacteriophages are the most abundant biological entities in existence [1]. They play essential roles in the ecology and genetics of bacteria and are in development as novel therapies for bacterial infections [2, 3]. However, lytic phages can have devastating consequences in modern industrial and scientific settings that depend on productive bacterial cultures, such as fermentation facilities and protein expression laboratories [4]. Once infections become entrenched, the acellular nature and general hardiness of bacteriophages make them less susceptible to sterilisation practices than other sources of microbial contamination [5]. Both natural products [6, 7] and synthetic polymers [8] have been identified that block phage infection, and the latter may be particularly useful as preventative culture additives due to their low cost and compatibility with bacterial growth. However, resolving established lytic outbreaks often depends on developing stable genetically resistant host strains through screening for spontaneously resistant mutants [9], carrying out targeted gene deletion/modification, or installing orthogonal elements of CRISPR-based acquired immunity systems [10].

The lytic phage infection cycle involves several steps, including adsorption via tail fibre and tip proteins to one or more receptors on the host cell, injection of its genetic material, synthesis and packaging of components to generate new virions, and finally lysis of the host cell to enable escape [11]. Any of these steps can be targeted to generate a rationally designed resistant host, although targeting adsorption by modifying or eliminating the phage receptor gene is the most frequently employed strategy [12]. This approach can be straightforward to implement, given knowledge of the relevant receptor. However, characterisation of bacteriophages lags that of their hosts, and the identity of most phage receptors remains unknown. In addition, much of the fine molecular details of the interaction between phage tail proteins and their receptors are poorly characterised, hindering the development of rational approaches to generate resistance without experimental characterisation. Such details are crucial if a bacteriophage targets an essential outer membrane protein, as generating a knockout of an essential gene will result in non-viability [12].

Bacteriophages that infect Gram-negative bacteria such as *E. coli* typically adsorb via interactions with lipopolysaccharide (LPS) and/or porin-like outer membrane proteins (OMPs) [13]. This is true of siphophages [14], with O-antigen glycans acting as a primary receptor and porins acting as the secondary and terminal receptor. A limited repertoire of porins have been reported to act as siphophage receptors, including FhuA, YncD, FepA, TolC, BtuB and LptD [13]. Of these, LptD is unique in that it is essential for viability in Gram-negative bacteria. LptD forms a 26-stranded β-barrel in complex with LptE positioned inside and functions to translocate lipopolysaccharide into the outer membrane [15–17]. LptD has been largely overlooked as a phage receptor, likely due to its essential nature, as many high-throughput and systematic attempts to identify novel receptors depend on gene disruption approaches that are amenable only to non-essential genes. However, two spontaneously generated mutants of LptD were recently reported that induced resistance to several small siphophages [18] apparently without compromising bacterial fitness. The mutations map to regions at or adjacent to extracellular loops, with the ΔL394-Y396::Y mutation resulting in the removal of a short section of β-strand 10 and the ΔY658-Y678::H deletion removing most of extracellular loop 11.

In this work we isolated a highly virulent, T1-like coliphage, provisionally named 7-West virus (7WV) after the location of the lab within our building, that caused rapid lytic infections of *E. coli* B cultures used for recombinant protein expression. This phage caused severe disruption to routine bacterial culture and proved intractable to elimination through surface cleaning and professional sterilisation. Genomic sequencing and morphological analysis by transmission electron microscopy (TEM) enabled classification of 7WV as a member of the siphophage family. Through comparative analysis of genome organisation and putative receptor binding proteins, we were able to identify it as a likely LptD-dependent virus and design and execute a strategy to successfully generate resistant *E. coli* strains that allowed our bacterial protein production activities to resume. To the best of our knowledge, this is the first rationally designed method to generate resistance to LptD-dependent lytic bacteriophages, and it should be broadly applicable to achieve resistance in any Gram-negative bacterial species.

## Results

### Isolation and Imaging of a Laboratory Bacteriophage Contaminant

**Fig. 1:**
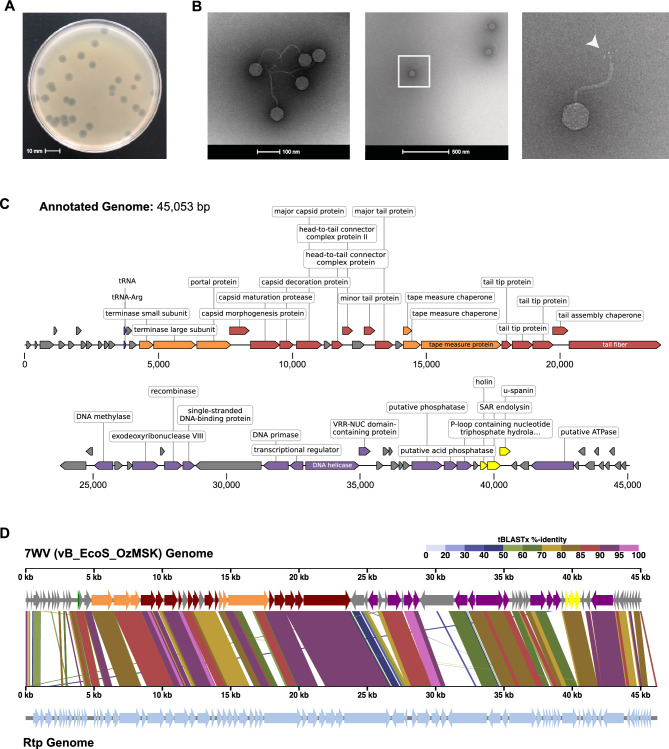
7WV/OzMSK Isolation and characterisation. **A** Double-layer agar plaque assay performed on sterile-filtered BL21(DE3) culture lysate at a dilution of 10-8 on the same host strain. **B** TEM images of CsCl gradient-purified, negative-stained phage particles. Boxed insert from the middle panel is enlarged in the right panel, with arrowhead indicating the tail tip structure. **C** Annotated genome of 45,053 bp with 44.2% GC content. 75 protein-coding genes and one tRNA were identified. Where protein functions can be inferred, these are indicated in boxed labels. **D** VipTree alignment with the Rtp genome illustrating high identity and synteny

7WV was isolated from lysed *E. coli* B-strain cultures used in routine recombinant protein expression. Filtered and diluted media from lysed cultures formed transparent plaques approximately 4–6 mm in diameter on double-layer agar plates (Fig. 1A) when co-inoculated with its bacterial host, BL21(DE3). Transmission electron microscopy (TEM) imaging of negatively stained 7WV (Fig. 1B) ruled out T1, instead revealing a siphophage with an icosahedral head of average diameter 60 nm (± 1.5 nm) and a long, non-contractile tail measuring 164 nm (± 5.3 nm) in length and 7.7 nm (± 1.3 nm) in diameter. The tail terminates with a rosette-like tail tip, akin to that observed in bacteriophage RTP [19]. The tails tended to curl back towards the head, likely due to the choice of ammonium molybdate as the fixative agent [20].

We carried out a limited screen to investigate the host range of 7WV (Table 1), with a particular interest in bacterial strains frequently used in routine molecular biology applications and recombinant protein expression. 7WV induced lysis in all B-strains tested, but it was unable to infect commercially sourced K-strains commonly used for recombinant DNA manipulation. Phage genomic DNA was subsequently prepared from double-layer agar plates displaying confluent lysis of BL21(DE3) *E. coli* and sequenced.

**Table 1:** Investigation of 7WV host range

Name of bacterial strain	Source	K- or B-derived	7WV Sensitivity
BL21(DE3)	NEB	B	+
C43(DE3)	Lucigen	B	+
C41(DE3)	Lucigen	B	+
Lemo21(DE3)	NEB	B	+
REL606	Non-commercial (Harms laboratory)	B	+
DH5α	NEB	K	–
XL1-Blue	Agilent	K	–
DH10B	NEB	K	–

### 7WV Genome Analysis

The assembled genome of 7WV (Fig. 1C) consisted of 45,053 bp with a GC composition of 44.2%. Alignment to previously reported phage genomes using BLASTN revealed that 7WV is closely related to other siphophages, including a set of 34 viruses (91.12–92.92%) isolated from clinical samples in Austria [21]; previously characterised phages ACG-M12 [22] (GenBank accession code NC_019404.1; 90.37% identity), IME253 [9] (NC_047810.1; 90.30%), Rtp [19] (NC_007603.1; 89.5%), SU57 [23] (MT511058.1; 89.13%) and DTL [10] (NC_047893.1; 85.37%); and members of the recently reported BASEL collection [18] AugustePiccard (MZ501051.1; 87.47%), JeanPiccard (MZ501080.1; 86.24%) and JulesPiccard (MZ501087.1; 86.11%). Sequence similarity, combined with the morphological evidence from TEM images, places 7WV within the *Drexlerviridae* family and *Braunvirinae* subfamily. Further investigation revealed that 7WV is near-identical genetically to bacteriophage MSK (only 9 nucleotides difference), which was recently isolated from a research laboratory in Hangzhou, China [24]—a match that did not appear in our initial searches because the GenBank entry for MSK (MW057918) is classified as unverified, excluding it from the compiled BLASTN databases. Hereafter, we refer to 7WV as vB_EcoS_OzMSK and we direct readers to the above report for further physical characterisation.

Gene annotation predicted 1 Arg tRNA gene and 75 open reading frames (ORFs), of which 34 were assigned a predicted function based on similarity to previously characterised phage genes (Fig. 1C and Supplementary Table 1). The order and size of the ORFs exhibited synteny with related siphophages such as Rtp [19] (Fig. 1D) and JeanPiccard [18] and fall into four broad categories: Nineteen ORFs code for proteins involved in phage assembly, forming the phage head and tail, binding to the terminal receptor and packaging DNA; Twelve ORFs code for proteins essential for metabolism and DNA replication; Three ORFs code for holin, endolysin and unimolecular spanin, which together form the machinery required for cell lysis. The remaining 41 ORFs are denoted as hypothetical proteins, as their function remains unknown. Though not thoroughly investigated here, the gene encoding one of these hypothetical proteins (NCBI accession no. XLM52761) was the site at which every raw contig of the vB_EcoS_OzMSK genome assembly was opened. Confirmatory sequencing of PCR products amplified in this region of the genome demonstrated variability in this gene, likely due to internal repeat sequences. Structural predictions of this 816 amino acid predicted protein revealed high similarity to multiple known phage tail spike proteins. The complete annotated vB_EcoS_OzMSK genome has been deposited with GenBank accession number PQ659294.

### Identification of the OzMSK Host Receptor

To develop a rational approach to creating resistant strains for laboratory use, we aimed to identify and disrupt the host receptor. However, the original MSK report [24] did not investigate this. We derived several spontaneously resistant clones by repeated culture on phage-coated agar plates, but these clones displayed slow growth kinetics in liquid culture and were therefore not likely to be useful for protein production. Furthermore, genome sequencing of several resistant BL21(DE3) clones revealed few shared mutations, and none in outer membrane proteins (Supplementary Tables 2 and 3), making it unlikely that resistance had arisen via alteration of the OzMSK terminal receptor.

In a systematic analysis of phage–host interactions recently reported by Maffei et al. [18], it was suggested that conservation of ORFs encoding RBPs was likely indicative of shared host receptor specificity, even among distantly related groups of small siphoviruses. The authors noted that two of the viruses for which they experimentally identified the terminal receptor as LptD (AugustePiccard and JeanPiccard) had highly homologous ORFs in similar genomic locations (immediately downstream of the *gpJ* homolog) to the bona-fide RBP locus of bacteriophage Rtp (exhibiting 93% nucleotide identity). As all three of these viruses were top hits in our BLASTN search with the OzMSK genome, we set out to test whether OzMSK also uses LptD as its terminal receptor and screened the two phage-resistant, LptD mutant strains identified by Maffei et al. [18] (kindly provided by the Harms laboratory) alongside their parental strain for OzMSK susceptibility. The parental strain K12 MG1655 ΔRM, a derivative of K12 MG1655 that lacks O-antigen glycans as well as all restriction enzymes and the RexAB and PifA abortive infection systems, was clearly susceptible to infection with OzMSK (Table 2). However, it was not able to infect either of the LptD-mutant K-12 strains, leading us to conclude that LptD is indeed the host receptor for bacteriophage OzMSK.

**Table 2:** Screen of LptD mutant host strains

Name of bacterial strain	Source	K- or B- derived	OzMSK sensitivity
K12 MG1655 ΔRM	Non-commercial (Harms laboratory)	K	+
K12 MG1655 ΔRM LptD ΔY658-Y678::H	Non-commercial (Harms laboratory)	K	–
K12 MG1655 ΔRM LptD ΔL394-V396::Y	Non-commercial (Harms laboratory)	K	–

### Evolutionary Analysis and Structural Modelling of the Putative LptD-Binding RBP

**Fig. 2:**
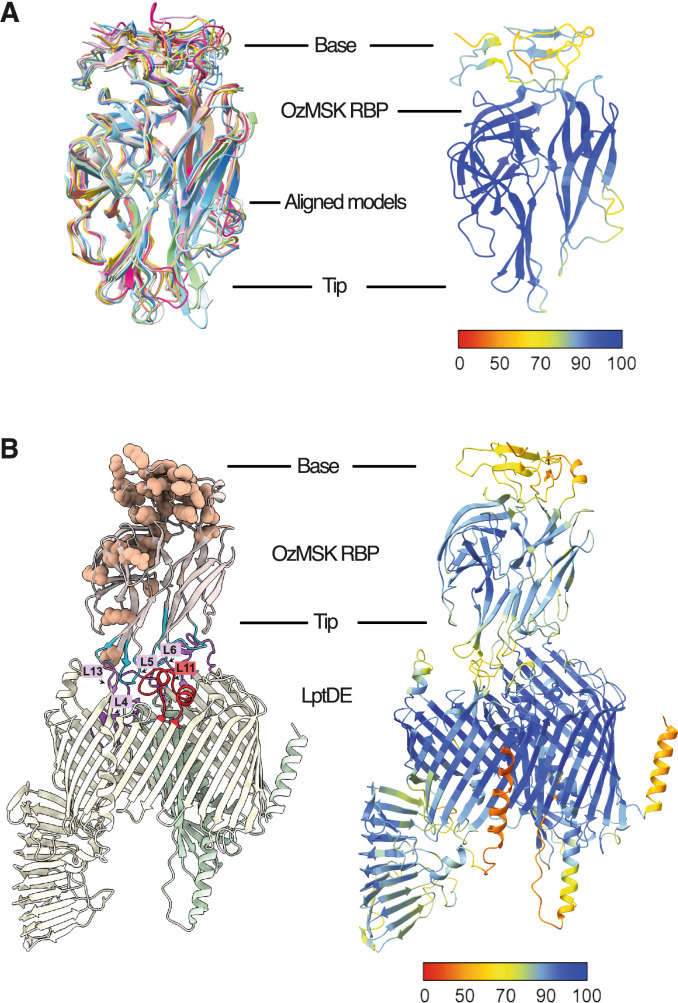
Sequence and structural analysis of putative LtpD-binding proteins from diverse phage genomes. **A** *Left:* Aligned AlphaFold2 generated models of predicted LptD-RBPs from diverse phages, including OekolAmpad (grey), BF9 (light blue), FritzHoffman (green), Rogue1 (hot pink), AugustePiccard (rose pink), SH6 (dark blue), IME542 (salmon), RTP (yellow) and 7WV/OzMSK (tan). *Right:* AlphaFold2 generated model of 7WV/OzMSK LptD-RBP coloured by pLDDT (confidence of prediction) score, with blue indicating high confidence, yellow moderate confidence and red low confidence. **B** AlphaFold3 generated model of a predicted complex between the 7WV/OzMSK LptD-RBP and *E. coli* LptDE. *Left:* Ribbon diagram illustrating the predicted interaction between LptD (pale yellow), LptE (pale green), and the RBP (pale rose pink). Conserved RBP residues across a multiple sequence alignment (MSA) of 16 diverse phages (Supplementary Figure 1) are displayed as tan space-filling spheres. Loops predicted to mediate interactions are highlighted in blue (RBP) and purple (LptDE). LptD loop 11 (red) is the site targeted for deletion. *Right:* Ribbon diagram of the predicted RBD: LptDE complex coloured by pLDDT (confidence of prediction) score, with blue indicating high confidence, yellow moderate confidence and red low confidence

The ORF encoding the putative RBP of OzMSK (CPT_vB_ecoS_OzMSK_038) has only moderate amino acid sequence identity to that of Rtp (39%), but a BLASTP search using the OzMSK_038 sequence returned more than a dozen hits with much higher (63.55–96.62%) amino acid identity (see Supplementary Figure S1). Many of these were predicted to be LptD binders in the above study [18]. To investigate the degree of structural homology among this group of proteins, we generated AlphaFold2 (AF2) [25] models of RBPs from predicted or experimentally validated LptD-dependent phages, across diverse families, and compared these with an AF2 model of the equivalent ORF from OzMSK. The OzMSK LptD-RBP model has significant structural conservation with the equivalent AF2-generated RBPs from other predicted or validated LptD-dependent phages (Fig. 2A), with pairwise RMSD values at approximately 1 Å or below (examples in Table 3). The predicted RBP structure is dominated by two lectin-like antiparallel β-sandwiches, creating a rigid scaffold with six loops extending from one end of the long axis (the tip) and a collection of loops and β-hairpins at the other end (the base). A DALI search revealed that the RBP has limited global homology with experimentally determined structures, with alignments predominantly occurring in the β-sandwich scaffold. The closest match was to a galactose-binding lectin (PDB ID: 3WMP), and weaker matches occurred with other phage tail fibre or tailspike proteins (PDB IDs: 4MTM, 6C15, 5JSD).

**Table 3:** Quality of structural alignment between OzMSK and other phage RBP models

Experimentally verified LptD-dependent phages			Predicted LptD-dependent phages			
AugustePiccard	FritzHoffman	OekolAmpad	Rogue1	SH6	RTP	BF9
Drexleviridae Braunvirinae	Queuovirinae Nonagvirus	Caudoviricetes Dhillonvirus	Drexleviridae Rogunavirinae	Drexleviridae Tunavirinae	Drexleviridae Braunvirinae	Caudoviricetes Dhillonvirus
0.785 Å RMSD to OzMSK	1.044 Å RMSD to OzMSK	1.073 Å RMSD to OzMSK	0.359 Å RMSD to OzMSK	0.84 Å RMSD to OzMSK	0.882 Å RMSD to OzMSK	1.050 Å RMSD to OzMSK

To investigate potential host receptor binding modes, we generated AlphaFold3 (AF3) [26] models of a complex between *E. coli* LptD, LptE and the OzMSK RBP (Fig. 2B). The LptDE complex within the AF3 models aligned very well with the corresponding parts of the experimentally determined LptDE crystal structure (PDB ID: 4RHB; pairwise RMSD 0.5 Å) and OzMSK RBP is predicted to bind via its pointed tip, with four of six RBD loops making extensive contacts to LptD extracellular loops 4, 5, 6, 7, 11 and 13. Interestingly, in our sequence alignment of 16 predicted or confirmed LptD-dependent phage RBPs (Supplementary Figure S1), the absolutely conserved residues map largely to the base and one side of the OzMSK RBP model rather than the predicted LptD-binding end (Fig. 2B). The base contains the N-terminus, reportedly involved in connecting the RBP to other tail proteins for many bacteriophages [27, 28], and this connectivity would appropriately orient the RBD towards the host membrane according to our model. Ordering the 16 RBP sequences by degree of conservation in the predicted LptD-binding loops (as in Supplementary Figure S1) defines three broad groupings that may utilise slightly different binding modes, but a more detailed analysis of specific contacts is limited by the moderate to low confidence (pLDDT) score of the AF3 model (< 70) in the loop regions.

### Generating a Targeted LptD Loop Deletion in BL21(DE3) Using CRISPR/Cas9 Templated Repair

**Fig. 3:**
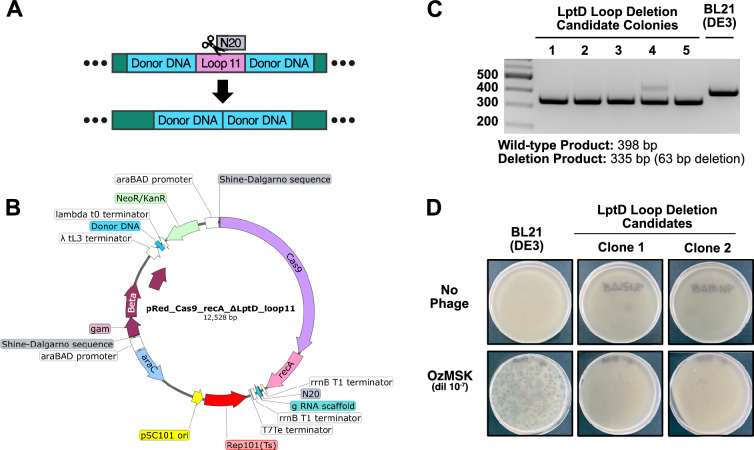
Generation of OzMSK-resistant *E. coli* BL21(DE3) through receptor engineering. **A** Schematic of targeting and repair sequences for genomic deletion of LptD Loop 11 Y658-Y678. These sequences were installed into the editing plasmid **B** in the indicated Donor DNA (cyan) and N20 (grey) sites. **C** PCR confirmation of successful gene editing (63-bp deletion) in 5 clones. PCR product from the parental BL21(DE3) strain is shown for comparison. **D** Double-layer plaque assay confirming OzMSK resistance in two representative clones

To create viable, OzMSK-resistant *E. coli* B strains for use in protein production, we sought a strategy to make a targeted deletion in LptD that would eliminate RBP binding. From a structural perspective, the ΔY658-Y678 loop 11 deletion identified by Maffei et al. [18] was more attractive than the ΔL394-V396 deletion that is situated in the middle of a β-strand of the outer membrane-embedded barrel structure. We therefore integrated targeting and repair template sequences (Fig. 3A) into the single-plasmid CRISPR/Cas9 system reported by Zhao et al. [29] that should delete LptD Y658-Y678, but without introducing the additional R679H substitution as in the spontaneous mutant [18]. This plasmid encodes the Cas9 protein and guide sequence under the *L*-arabinose-inducible pBAD promoter, along with repair template DNA and ƛRed recombinases for editing efficiency. It also carries a temperature-sensitive origin of replication for simple curing after successful editing (Fig. 3B).

Complete details of the targeted deletion protocol are provided in *Materials and Methods* and briefly summarised here. BL21(DE3) was transformed with the plasmid pRed_Cas9_recA_∆LptD_loop11 (available on request) and then plated onto Kanamycin-containing LB agar and grown overnight at 30 °C. A single colony was grown in liquid culture under arabinose induction and then plated on Kan^+^ arabinose^+^ LB agar and grown overnight again at 30 °C. Colonies were screened by PCR (Fig. 3C) for the 63 bp deletion, confirmed by Sanger sequencing, and then cured of the plasmid by growth in non-selective medium at 37 °C (see Methods). A final set of cured clones was reconfirmed by PCR and tested for susceptibility to lysis by OzMSK alongside the parental BL21(DE3) strain (Fig. 3D). One clone was selected to make a chemically competent glycerol stock for testing of growth kinetics and high-yield expression of recombinant proteins.

### Analysis of BL21(DE3) LptD ΔY658-Y678 Strain Growth and Protein Expression

**Fig. 4:**
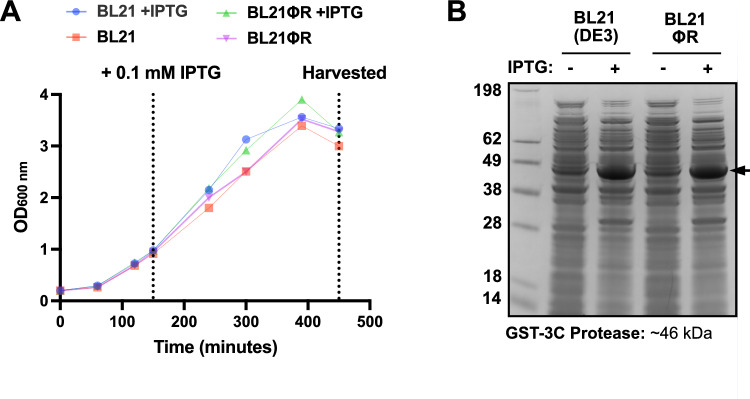
BL21(DE3) OzMSK resistant (R) strain growth and protein expression analysis. Parental and phage-resistant BL21(DE3) were transformed with pGEX-2T-GST-3C-Protease and inoculated from overnight starter cultures into fresh LB with 30 g/ml kanamycin and monitored over ~8 hours. Growth **A** and GST-3C protease **B** expression were monitored before and after induction with IPTG. Coomassie stained SDS-PAGE gel shows equivalent IPTG-induced 3C protease (*arrow*) expression in parental and R whole cell lysates

To be useful in the laboratory, it is essential that the OzMSK-resistant BL21(DE3) strain maintains both rapid growth kinetics and good recombinant protein expression yields. To investigate this, we compared the growth of mutant phage-resistant (ΦR) and parental BL21(DE3) in rich broth and found their growth rates to be indistinguishable over 8 h (Fig. 4A). We further compared recombinant protein expression using a GST-human rhinovirus 3C protease-encoding plasmid with IPTG induction and again found indistinguishable bands at the expected molecular weight of the target protein (Fig. 4B). Our laboratories are now using BL21(DE3)ΦR as our primary protein expression strain with good protein yields and no lysed cultures.

## Discussion

Reports of novel bacteriophages often provide extensive analysis of their genome, host range and morphology but fail to identify the host receptors they exploit. Predicting these receptors using computational methods can be difficult due to extensive horizontal gene transfer and a lack of phenotypic data linking genetic sequences to specific receptor binding [27, 30]. Here, we isolated a laboratory contaminant coliphage and provided experimental and structural evidence that it exploits LptD to adsorb to BL21(DE3) *E. coli*. It has been previously reported that a conserved RBP ORF is shared by LptD-dependent phages [18] and this ORF is present in the OzMSK genome, although the overall sequence identity compared to other experimentally verified LptD-dependent phages is low. This is perhaps unsurprising, given the rapid mutation rate involved in viral replication, with selective pressure acting to maintain only sequences essential for the RBP to assemble into the phage tail and interact with LptD. Understanding which regions of the RBP are responsible for these functions and defining their associated sequence conservation signatures may therefore have predictive power.

The AlphaFold models of OzMSK RBP in complex with LptDE, together with our sequence alignment with other likely LptD-binding RBPs, allow speculation on the interactions that mediate assembly and phage absorption. It is likely that LptD binding involves contacts between the extended array of loops that project from the tip of the RBP and several extracellular loops (4, 5, 6, 7, 11 and 13) of LptD. This model is consistent with our successful generation of OzMSK resistance via a targeted LptD loop 11 deletion, and it is further supported by the recent report of at least one other coliphage for which missense mutations in LptD loops 4 and 11 conferred resistance [31]. This type of loop-mediated contact is a common feature of phage–receptor interactions, as seen in the recent cryoEM structures of the T5 RBP pb5 in complex with its host receptor FhuA [32] and of the bacteriophage lambda RBP gpJ bound to its host receptor LamB [33]. The OzMSK RBD-LptDE model also implies that RBP surfaces where sequence conservation is most concentrated are likely to mediate assembly into the phage tail rather than host receptor binding, and we suggest that some combination of these conserved sequences and the LptD-contacting loops identified here will provide a useful predictor of LptD dependency in newly isolated coliphages.

Identification of our Melbourne, Australia-based laboratory contaminant bacteriophage as genetically near-identical to one recently isolated in Hangzhou, China [24], underscores the ubiquitous and robust nature of lytic phages and the ease with which they can rapidly become paralysing challenges, even across large geographic distances, for biomolecular production facilities that rely on bacterial propagation. The physical characterisation provided by Khan et al. [24] indicated extreme stability of MSK in a range of harsh conditions, including pH (stable 4–12), temperature (stable > 100 °C) and UV exposure. Its short latent period (25 min) and large burst size (> 200 virions per cell) in their analysis further validate our experience of MSK as a formidable laboratory pest. The essential nature of the host receptor protein LptD presented a challenge to production of resistance by simple gene disruption but, fortunately, the study that led to our identification of LptD as the likely receptor [18] also provided a solution in the form of specific mutations that could be replicated using a simple gene-editing strategy [29]. Our results indicate that Cas9-mediated deletion of LptD loop 11 does not negatively impact the overall growth kinetics of BL21(DE3) at 37 °C or its utility for recombinant protein production. This aligns with other reports that deletion of loop 11 supports wildtype-like growth kinetics [34] and fitness [18] in K-12 *E. coli*, and it indicates that our approach should be widely applicable. Nonetheless, there are examples in bacteriophage evolution that indicate acquisition of more than one outer-membrane protein as terminal receptor [35] or even shifting of RBP loop-binding preferences on the same receptor [36], making the permanence of any receptor engineering approach far from guaranteed. It is possible that introduction of heterologous defence systems based on engineered bacterial exclusion (BREX) [37, 38] or CRISPR/Cas9 [10, 39] islands could provide broader or more durable immunity than targeted receptor modification. Whether these approaches have fitness costs or benefits compared to loop 11 deletion for protection against OzMSK or other LptD-dependent phages would need to be tested experimentally and may be application dependent.

In conclusion, to the best of our knowledge this is the first report of a rationally designed approach to generate resistance to bacteriophage that uses the essential LPS transporter protein LptD as a terminal receptor. This method leverages an efficient single-plasmid system designed for rapid *E. coli* genome editing [29] to provide an alternative to previously reported methods that use orthogonal CRISPR systems [10], lambda red recombinase [40] or generation of resistance spontaneously [9]. Furthermore, our structural and bioinformatic analysis of the LptD-RBP ORF builds on the recent work of Maffei et al. [29] to enable further in silico identification of LptD-dependent bacteriophage, potentially enhancing characterisation of phage–host interactions and supporting the development of phage-resistant bacterial strains for research and industrial use.

## Materials and methods

### Bacterial Strains and Growth Conditions

The parental BL21(DE3) *E. coli* strain was from NEB (Cat# C2527) and contained the T1-resistant *fhuA2* allele. All *E. coli* strains were grown in LB medium with aeration (180 rpm) at 37 °C and on LB agar at 37 °C unless otherwise stated. Deletion mutants were selected using kanamycin at 30 µg/ml.

### Soft-Agar Plaque Assay

A sample of fresh overnight liquid culture (100 μl) of the indicator strain was mixed with an equal volume of sterile-filtered supernatant from lysed BL21(DE3) culture (titer ~ 3 × 10^10^ pfu/ml) diluted in PBS containing 1 mM CaCl_2_ and 1 mM MgSO_4_ on ice and then warmed to 30 °C in a water bath for 5 min to initiate synchronised infection. Each sample was then mixed with 3 ml molten LB soft agar (0.75%) and poured onto room temperature LB hard agar (1.5%) plates. Phage plaques were observed and enumerated after 18–24 h of incubation at 37 °C.

### Phage Genome Purification

Phage lysates were prepared from 90 mm double-layer LB-agar plates at confluent lysis by extracting into LB medium, sterile filtering (0.22 μm pore size) and precipitating in 10% PEG-8000, 1 M NaCl overnight at 4 °C. Precipitated phage particles were collected by centrifugation (10,000 g for 30 min) and resuspended in 5 mM MgSO_4_. A 500 μl aliquot was treated with 12.5 U DNase I and 25 μg RNase A (1 h at 37 °C) to eliminate bacterial genetic material. This digest was quenched by the addition of 20 mM EDTA and phage capsids were subsequently digested with 25 μg proteinase K in 0.5% SDS (1 h at 60 °C) to release phage DNA. The digest reaction was then phenol:chloroform extracted and the aqueous phase was salt:ethanol precipitated to collect phage genomic DNA, which was resuspended in 10 mM Tris with 1 mM EDTA (pH 8.0) and analysed for purity and integrity by agarose gel electrophoresis.

### Phage Genome Sequencing

Illumina TruSeq Nano low-throughput kit was used to prepare a library for Illumina MiSeq sequencing with 250-bp paired-end reads using a 500-cycle v2 kit. Quality control of 333,456 total sequenced reads was performed using FastQC (http://www.bioinformatics.babraham.ac.uk/projects/fastqc). After trimming was performed using the FastX Toolkit v0.0.14 (http://hannonlab.cshl.edu/fastx_toolkit), the raw 7WV contig was assembled using SPAdes v3.5.0, with default parameters including k-mers of 21, 33 and 55, to 557-fold coverage [41]. A second contig, assembled using adaptive k-mers, was opened in a different place, allowing complete closure of the genome sequence. Despite this, PhageTerm analysis with raw sequencing reads was unable to assign the terminus type [42]. Assembly and ends analysis were performed on the Center for Phage Technology Galaxy instances (https://phage.usegalaxy.eu/) [43], Pharokka (https://github.com/gbouras13/pharokka) [44] and Phold (https://github.com/gbouras13/phold), accessed through a Colab Notebook. Similarity searches were performed using BLASTN with default parameters on the local Galaxy instance [45]. Alignment map with Rtp was generated using VipTree4.0 with the standard input parameters [46].

### Intact Phage Particle Preparation

PEG-precipitated phage suspension (1.6 ml) was first chloroform extracted to remove residual PEG and cell debris. The aqueous phase was then added to solid CsCl (0.5 g/ml), mixed gently to dissolve and layered onto a discontinuous gradient of 2 ml CsCl at 1.70 g/ml, 1.5 mL at 1.50 g/mL and 1.5 mL at 1.45 g/mL in a Beckman SW41 ultracentrifuge tube. Gradients were developed at 22,000 rpm for 2 h at 4 °C. The visible phage particle band was collected by syringe and dialysed overnight against 100 mM NaCl, 8 mM MgSO_4_, 10 mM Tris pH 7.5 at to 4 °C remove CsCl. The final phage preparation was stored at 4 °C until required for imaging.

### Transmission Electron Microscopy (TEM)

Phage suspension at a concentration of 3 × 10^6^ particles/μl was applied to a glow-discharged, carbon-coated copper grid (300 mesh). After 30 s, the solution was blotted off and the grid was washed in water and stained with 2% ammonium molybdate (pH 7) for 30 s. Excess solution was again blotted off and the grid was imaged at room temperature using a Talos L120C transmission electron microscope with 70 μm aperture at a defocus value of approximately −1 um.

### Identification of Variants Associated with Spontaneously Resistant *E. coli* Genomes

Short read sequencing data quality was checked using fastqc (http://www.bioinformatics.babraham.ac.uk/projects/fastqc/). Initial alignment of the sequencing data was performed using bwa [47] and bbmap [48] to several *E. coli* reference genomes, including BL21DE3 (GenBank accession code CP001509.3), C43DE3 (CP011938.1), O127:B8 C43/90 (NZ_AHAW00000000.1) and HUSEC2011 (HF572917.2). SNVs/indels were generated by bbmap callvariants.sh. Copy numbers were called using sequenza [49] and cnvkit [50]. SVs were called using by GRIDSS [51, 52]. Adapter trimming and deduplication were performed for bbmap alignments. Alignment metrics were collected using picard tools (http://broadinstitute.github.io/picard) and variant counts compared. Based on its high mapping rate and low number of structural variants, BL21DE3 (CP001509.3) [53] was selected for final analyses. Variants associated with phage-resistant *E. coli* genomes were identified by comparison with contemporaneously sequenced sensitive (preselection) genomes. Analysis code is available at https://github.com/PapenfussLab/ecoli_bacteriophage.

### Identification and Structural Analysis of Putative RBPs and RBP-LptD Interactions

Putative LptD-RBP domains were identified from the genomes of both experimentally verified LptD-dependent phages (AugustePiccard, FritzHoffman, OekolAmpad, RTP) and predicted LptD-dependent phages (IME542, Rogue1, SH6, and BF9). The ORF was selected based on conserved relative position downstream of the tail fibre protein and a consistent ORF length of 930–960 bp. The DNA sequences were extracted and translated into amino acid sequences. Protein structure predictions of the RBPs alone were carried out with ColabFold (version 1.3.0), an implementation based on AlphaFold2 [25], through the Google Colab user interface. No templates or additional relaxation were used for predictions. Alignments were generated with the MMSeqs2 [54] (UniRef + Environmental) MSA server with three recycles during prediction. Protein structure predictions of RBP-LptD complexes were generated using the AlphaFold3 [26] server (https://alphafoldserver.com/), version 3.0.0. In all cases, five models were generated per sequence. Models were ranked by predicted local distance difference test (pLDDT) scores and confidence scores were assessed to evaluate model quality. The structure with the highest pLDDT score for each sequence was selected for structural alignment in ChimeraX [55], where root-mean-square deviation (RMSD) values were calculated to assess structural similarities. No additional refinement of structures was performed post-prediction.

### CRISPR Plasmid Design and Editing

The editing plasmid pRed_Cas9_recA_∆poxb300 was obtained from MolecularCloud (#MC_0000001) and used as a template for amplification and assembly of the LptD loop deletion plasmid as per the original paper [29]. Oligos for amplification of segments of the plasmid, annealing oligos for assembly of the LptD-targeting N20 guide sequence, annealing oligos for assembly of the LptD loop 11 deletion donor DNA and plasmid assembly verification primers (Supplementary Table 4) were obtained from IDT. The final editing plasmid, pRed_Cas9_recA_∆LptD_loop11, was assembled using the NEBridge Golden Gate Assembly Kit (Bsa-HF v2) (NEB cat# E1601S) and verified via Nanopore sequencing at the WEHI Advanced Genomics Facility. The entire plasmid sequence is provided as a supplementary SnapGene file.

For genome editing, pRed_Cas9_recA_∆LptD_loop11 plasmid was transformed into NEB BL21(DE3) (cat# C2527) cells, plated onto Kan^+^ selection medium and incubated at 30 ℃ over two days, then stored at 4 ℃. On the third day, a single colony was inoculated into 5 ml LB + Kan and expanded over 5 h at 30 ℃ (250 rpm) before gene-editing was induced via the addition of 2 g/L arabinose. Cells were grown for another 4 h at 30 ℃ and 250 rpm, then collected by centrifugation and plated onto LB Kan^+^ arabinose^+^ selection plates and incubated overnight at 30 ℃. Successful deletion of loop 11 of LptD was confirmed through single-colony PCR (see below) and subsequent Sanger sequencing of the amplicon, after which the strain was cured of the heat-sensitive pRed_Cas9_recA_∆LptD_loop11 plasmid via overnight culturing at 37 ℃ with no antibiotic.

### PCR Analysis of LptD ΔY658-Y678 BL21(DE3)

LptD deletion candidate colonies were screened by single-colony PCR using verification primers (Supplementary Table 3.3) and Q5 polymerase to amplify the target region. The PCR products were then resolved on a 3% agarose gel to identify potential candidates. To confirm the identity of the PCR products, gel bands were excised and purified using the Zymoclean Gel DNA Recovery Kit (Zymo Research #D4008), followed by Sanger sequencing at the Australian Genome Research Facility (AGRF).

### Growth and Protein Expression Analysis

*E. coli* cells were transformed with the expression vector pGEX-2 T-GST-3 C-Protease and cultured in LB medium containing 100 µg/ml ampicillin. After overnight incubation, the culture was expanded at 37 °C and 180 rpm, until an OD_600_ of approximately 0.9 was reached. The culture was then cooled to 30 °C, and protein expression was induced by the addition of 0.1 mM isopropyl *β*-D-thiogalactopyranoside (IPTG). Cultures were grown for an additional 5 h before harvesting. OD_600_ measurements were taken every 30–60 min to monitor growth, and samples were collected pre-induction and at harvest for analysis. For protein expression analysis, whole cell lysates were prepared by boiling and sonicating cells in the presence of 1 mM DTT. The lysates were then separated on 12% NuPAGE Bis–Tris gels (Thermo Fisher Scientific) at 200 V for 35 min, and proteins were visualised using InstantBlue Coomassie Protein Stain (Abcam).

## Supplementary Information

Below is the link to the electronic supplementary material.

Supplementary file1 (XLSX 13 kb)

Supplementary file2 (PDF 3074 kb)

Supplementary file2 (DNA 3074 kb)

## Data Availability

The genome sequence and associated data for phage vB_EcoS_OzMSK were deposited under GenBank accession no. PQ659294, BioProject accession no. PRJNA222858, Sequence Read Archive accession no. SRR31488468 and BioSample accession no. SAMN45033504.
