# Supplementary material for: Resolution of a T1-Like Bacteriophage Outbreak by Receptor Engineering
Source: Mol Biotechnol. 2025 Jun 3;68(3):1411–23. doi: 10.1007/s12033-025-01453-1 (PMC13035648; doi:10.1007/s12033-025-01453-1)
Supplement: Supplementary file 2 — Supplementary file2 (PDF 3074 kb) [file 12033_2025_1453_MOESM2_ESM.pdf]

Supplementary Table 1. 7WV/vB_EcoS_OzMSK genome annotation table. See Supplementary Material file OzMSK_Annotation.xlsx.

Supplementary Table 2. Protein alterations that arose in spontaneously resistant BL21(DE3) clones.

	**BL21(DE3)**	
**Gene**	**Pre-selection**[#]	**Post-selection**[§]
YadC		K^{410}->N
	D^{413}->H	H^{413}->Y
		D^{427}->N
	G^{428}->*	$*^{428}$->R
		T^{429}->A
	P^{431}->T	T^{431}->*
		I^{432}->L
	E^{433}->K	K^{433}->Q
YadK	N^{140}->H	H^{140}->D
	N^{158}->H	H^{158}->D
	S^{165}->L	L^{165}->*
	A^{166}->R	R^{166}->L
	N^{168}->K	K^{168}->R
		S^{169}->T
	Y^{173}->D	D^{173}->Y
	T^{177}->N	N^{177}->I
	S^{178}->F	F^{178}->G
AGP	Mobile element excision disrupted C-terminus: `AANLLI*`→`DLFLQVMI*`	

[#]Indicates variants that were present in our lab BL21(DE3) strain compared to reference genome CP001509.3 without selection. [§]Indicates variants that were present in at least one clone compared to pre-selection genome after repeated culture in the presence of 7WV.

Supplementary Table 3. Protein alterations that arose in spontaneously resistant CD43(DE3) clones.

	C43(DE3)	
**Gene**	**Pre-selection#**	**Post-selection§**
sltY		S^{35} -> I
creA		S^{142}->P
	N^{145}->I	I^{145}->T
creB	L^{229}->P	P^{229}->L
creC	R^{2}->C	C^{2}->S
		L^{7}->*
		E^{394}->D
		D^{413}->E
creD		N^{135}->K
yjjX		I^{79}->M
	V^{89}->A	A^{89}->E
		S^{152}->R
cydA	E^{99}->G	G^{99}->A
	I^{98}->K	K^{98}->T
	E^{99}->G	G^{99}->S
		G^{99}->A
		L^{101}->V
		M^{102}->*

	C43(DE3)	
**Gene**	**Pre-selection#**	**Post-selection§**
ettA		R^{146}->S
	A^{150}->P	P^{150}->S
	D^{155}->H	H^{155}->N
	K^{267}->M	M^{267}->R
		I^{269}->R
		E^{270}->K
		K^{271}->S
		E^{272}->W
		L^{273}->S
		E^{274}->G
		W^{275}->Y
		Q^{278}->E
		G^{279}->*
trpR	K^{72}->*	$*^{72}$->Q
hyaA	Deletion/frameshift<br>^{63}ICCTESFIRSAHPLAKDVILS→<br>TVPNLLSAPLTHWRRTSSFP*	

#Indicates variants that were present in our lab C43(DE3) strain compared to reference genome CP011938 without selection. §Indicates variants that were present in at least one clone compared to pre-selection genome after repeated culture in the presence of 7WV.

Supplementary Table 4. Primers for assembly and verification of the CRISPR deletion plasmid and verification of the LptD loop 11 deletion.

**3.1 Plasmid Assembly Primers**		
**Primer**	**Sequence**	**Description**
Part1_F	CCAGGTCTCAGCTCTGCTGAATGGAAGCTTGGAT TCTCACC	Part 1 Amplification Primer F
Part1_R	CCAGGTCTCACGCTTAAGATCTGACTCCATAACA GAGTACTCGCC	Part 1 Amplification Primer R
Part2_F	CCAGGTCTCAGTTTTAGAGCTAGAAATAGCAAGT TAAAATAAGGC	Part 2 Amplification Primer F
Part 2_R	CCAGGTCTCAGCACCACAGGCCCATGGATTCTTC G	Part 2 Amplification Primer R
LptD_Del_Donor-F	GTGCGGCCAGCTGGCGACAGCACCTACCTGCGA AATACCATTCTTACGGTAATTCAGCTGTACCAGA CGGTCTTCATCCCGACGGT	Donor DNA Annealing Oligo F
LptD_Del_Donor-R	GAGCACCGTCGGGATGAAGACCGTCTGGTACAG CTGAATTACCGTAAGAATGGTATTTCGCAGGTAG GTGCTGTCGCCAGCTGGCC	Donor DNA Annealing Oligo R
LptD_N20_F	AGCGAGCGTAGCCTGAATATATTC	N20 Annealing Oligo F
LptD_N20_R	AAACGAATATATTCAGGCTACGCT	N20 Annealing Oligo R
**3.2 Plasmid Assembly Verification Primers**		
**Primer**	**Sequence**	**Description**
pkd_cas9_0033F	GTGAAGGCCTGCATTATGTC	Donor DNA Ligation Verification Primer F
pkd_cas9_0033R	CTCCACTTGCGTTAATAGGG	Donor DNA Ligation Verification Primer R
0033_N20_F	AAGTACAGACAGGCGGATTC	Donor N20 Ligation Verification Primer F
0033_N20_R	CCTACCTACGTAACGGACTAAG	Donor N20 Ligation Verification Primer R
**3.3 LptD Loop 11 Deletion Verification Primers**		
**Primer**	**Sequence**	**Description**
LptD-Del-F-Check	CGAATTGCATAGCAGCAGG	Checks for loop deletion
LptD-Del-R-Check	TGGGAGAATGACGACAAAAC	Checks for loop deletion

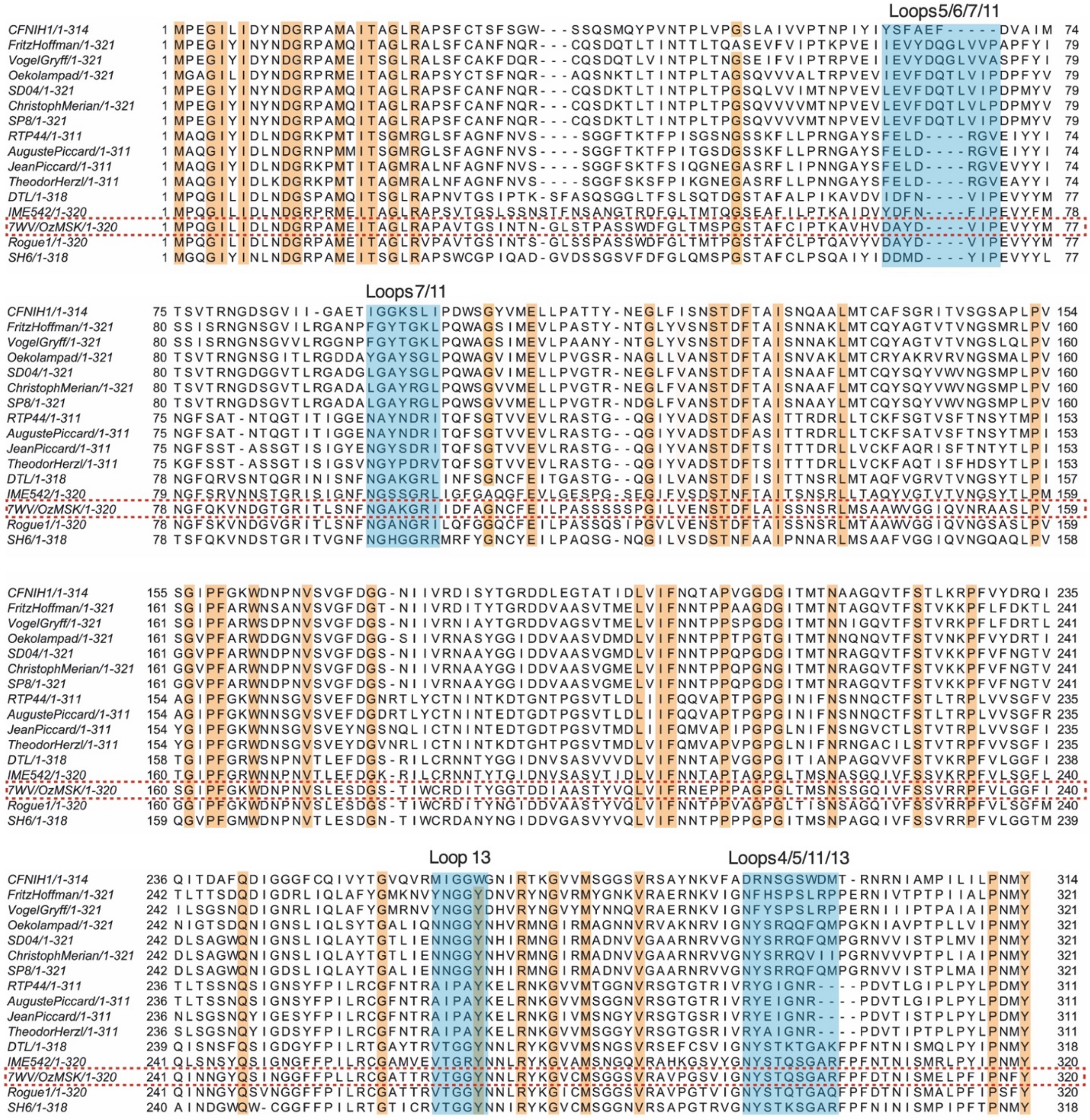

Supplementary Figure 1. Alignment of likely RBP sequences from predicted or experimentally validated LptD-dependent bacteriophages genomes. 7WV/OzMSK RBP sequence is boxed in red dotted lines. Phage designation and amino acid numbers are given in labels to the left. Conserved amino acids are highlighted in light orange. Sequences predicted to contact LptD are highlighted in light blue with their likely LptD loop contacts indicated above. Entries are arranged to emphasize subgroups based on conservation of LptD-contacting loop sequences.
